# The Wonderful Activities of the Genus *Mentha*: Not Only Antioxidant Properties

**DOI:** 10.3390/molecules26041118

**Published:** 2021-02-20

**Authors:** Majid Tafrihi, Muhammad Imran, Tabussam Tufail, Tanweer Aslam Gondal, Gianluca Caruso, Somesh Sharma, Ruchi Sharma, Maria Atanassova, Lyubomir Atanassov, Patrick Valere Tsouh Fokou, Raffaele Pezzani

**Affiliations:** 1 Department of Molecular and Cell Biology, Faculty of Basic Sciences, University of Mazandaran, Babolsar 4741695447, Iran; m.tafrihi@umz.ac.ir; 2 University Institute of Diet and Nutritional Sciences, Faculty of Allied Health Sciences, The University of Lahore, Lahore 54600, Pakistan; mic_1661@yahoo.com (M.I.); tabussamtufail@gmail.com (T.T.); 3 School of Exercise and Nutrition, Deakin University, Victoria 3125, Australia; tgondal@deakin.edu.au; 4 Department of Agricultural Sciences, University of Naples Federico II, 80055 Portici (Naples), Italy; 5 School of Bioengineering & Food Technology, Shoolini University of Biotechnology and Management Sciences, Solan 173229, India; someshsharma@shooliniuniversity.com (S.S.); mails4sharmaruchi@gmail.com (R.S.); 6 Scientific Consulting, Chemical Engineering, University of Chemical Technology and Metallurgy, 1734 Sofia, Bulgaria; 7 Saint Petersburg University, 7/9 Universitetskaya Emb., 199034 St. Petersburg, Russia; lyubomir.atanassov@gmail.com; 8 Department of Biochemistry, Faculty of Science, University of Bamenda, Bamenda BP 39, Cameroon; 9 Department of Biochemistry, Faculty of Science, University of Yaoundé, NgoaEkelle, Annex Fac. Sci., Yaounde 812, Cameroon; 10 Phytotherapy LAB (PhT-LAB), Endocrinology Unit, Department of Medicine (DIMED), University of Padova, Via Ospedale 105, 35128 Padova, Italy; 11 AIROB, Associazione Italiana per la Ricerca Oncologica di Base, 35128 Padova, Italy

**Keywords:** mint, *Mentha*, phytochemicals, essential oils, anticancer, antimicrobial

## Abstract

Medicinal plants and their derived compounds have drawn the attention of researchers due to their considerable impact on human health. Among medicinal plants, mint (*Mentha* species) exhibits multiple health beneficial properties, such as prevention from cancer development and anti-obesity, antimicrobial, anti-inflammatory, anti-diabetic, and cardioprotective effects, as a result of its antioxidant potential, combined with low toxicity and high efficacy. *Mentha* species are widely used in savory dishes, food, beverages, and confectionary products. Phytochemicals derived from mint also showed anticancer activity against different types of human cancers such as cervix, lung, breast and many others. Mint essential oils show a great cytotoxicity potential, by modulating MAPK and PI3k/Akt pathways; they also induce apoptosis, suppress invasion and migration potential of cancer cells lines along with cell cycle arrest, upregulation of Bax and p53 genes, modulation of TNF, IL-6, IFN-γ, IL-8, and induction of senescence phenotype. Essential oils from mint have also been found to exert antibacterial activities against *Bacillus subtilis*, *Streptococcus aureus*, *Pseudomonas aeruginosa*, and many others. The current review highlights the antimicrobial role of mint-derived compounds and essential oils with a special emphasis on anticancer activities, clinical data and adverse effects displayed by such versatile plants.

## 1. Introduction

Medicinal plants and their derived compounds (phytochemicals) have been considered of pharmacological significance since ancient times. The use of plants in medicine dates back to 60,000 years ago, before the birth of civilization [1]. Today, more than 30% of all medicinal drugs (and their derivatives and analogs) derive from plants, and natural products will continue to possess considerable impact in human medicine. Most synthetic bioactive drugs are structurally similar to the phytochemicals of plants from which they were firstly isolated [2,3]. In many developing countries, plant materials have an important role in primary care or disease treatment. In addition, due to contraindications in the usage of chemical drugs, there is a growing interest in the utilization of the plant-derived medicinal products [4], since compared to synthetic drugs some of these natural products have lower toxicity and higher efficacy [5]. Anticancer drugs, antibiotics, anti-inflammatory drugs, immunomodulators are among the most important drugs having herbal origin [6,7,8]. Moreover, some of the aforementioned plant-derived compounds have pleasant taste and odor and can be used in kitchen as flavorings, spices and food [9]. Among the plants with global economic and culinary importance, mint is used worldwide for perfuming sweet and savoury dishes and flavouring tea, in addition to its pharmacological importance.

## 2. *Mentha* Genus

*Mentha* is a genus belonging to the family of Lamiaceae, whose plants are among the most aromatic and spread in diverse environments worldwide [10], having simple, characteristic leaves with pleasant scent. *Mentha* taxonomy is highly complicated and includes about 42 species and 15 hybrids, with hundreds of subspecies and cultivars [11]. Eleven naturally occurring hybrids have been produced from the species *M. arvensis* L., *M. aquatica* L., *M. spicata* L., *M. longifolia* L., and *M. suaveolens* Ehrh; most hybrids are infertile but can propagate due to their highly invasive rhizome [12]. Plants of this genus are perennial and are used for essential oil production, mainly in USA, India, China, and Iran [13]. Fresh and dried plant materials of *Mentha* species are widely used in industry as part of confectionaries, flavor enhancing agents, pharmaceuticals, cosmetics, etc. [14]. Some *Mentha* species have common names, as listed in Table 1 [15,16,17].

## 3. Phytochemical Composition of *Mentha*

*Mentha* species are rich in polyphenols [18] and, moreover, contain caffeic acid and its derivatives caftaric acid, cinnamic acid, ferulic acid, and oleanolic acid [19,20,21]. Flavonoids including luteolin and its derivatives apigenin, acacetin, diosmin, salvigenin and thymonin, have also been detected in these plants, accounting for some 10–70 compounds out of the total phenolics, and also flavanols such as catechin, epicatechin and coumarins, including esculetin and scopoletin [22,23,24,25]. With regard to mint compositions, the essential oils represent a major focus. They are colorless, pale yellow or greenish yellow [26] and alcohols, ketones, esters, ethers and oxides are their main components in *Mentha* species [13,27]. Menthol, menthone, isomenthone, menthyl acetate, linalool, linalyl acetate, lippione, pulegone, carvone, piperitenone oxide and *cis*-piperitone epoxide are the main constituents of essential oils prepared from different mint species [13,28,29,30]. Table 2 lists the main compounds isolated from different species of this genus.

## 4. Properties of the *Mentha* Genus

*Mentha* species are characterized by a great chemical diversity and were reported to contain a number of chemical compounds responsible for pharmacological properties (Table 3).

**Figure 1:**
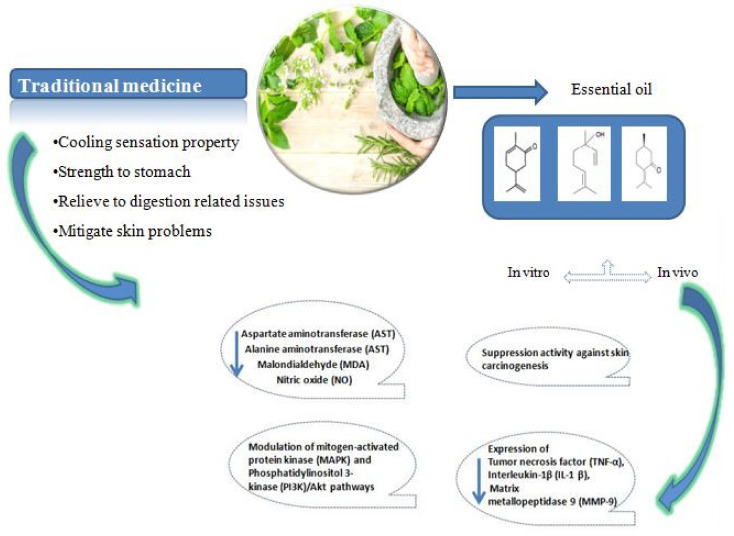
Summary of the main effects of *Mentha* species.

The use of essential oils has a long history being widely exploited in the food, beverage, confectionery and cosmetic industries. In traditional Iranian medicine, it is reported that *Mentha* species have cooling sensation properties, strengthen the stomach and are effective to relieve digestive symptoms, respiratory tract problems and hemorrhoids [72]. It has been shown that *Mentha spicata* essential oil can reduce pain caused by Caesarean section [73,74]. Recent investigations have suggested that the transient receptor potential cation channel subfamily M (melastatin) member 8 (TRPM8) and transient receptor potential cation channel, subfamily A, member 1 (TRPA1) are implicated in pain relief (analgesia) and sensation of cooling [73,75]. In Ayurvedic medicine, some *Mentha* species were used to mitigate skin problems and headaches [76]. In vitro studies showed that peppermint essential oil and menthol acted as smooth muscle relaxants via blocking Ca^2+^ influx through L-type Ca^2+^ channels [77,78]. Also peppermint juice led to reduction in total cholesterol levels, triglycerides and could increase HDL levels in the blood of university students [79] (Figure 1).

### 4.1. Antioxidant Activities

There is increasing interest in the utilization of plant-based natural antioxidants due to their safer nature and medicinal benefits compared to synthetic formulations. In this regard, extracts and essential oils (EOs) from many medicinal and food herbs have been investigated as promising source of effective antioxidant agents [80] showing a well-known action against reactive oxygen species and free radicals. A range of in vitro antioxidant assays such as (2,2-diphenyl-1-picrylhydrazyl) (DPPH) radical scavenging [81], 2,2’-azino-bis(3-ethylbenzothiazoline-6-sulfonic acid) (ABTS+) inhibition of linoleic acid peroxidation [82], and reducing power [83] assays have been successfully employed by the researchers to examine the antioxidant effects of *Mentha* plants. Numerous medicinal plants, including the *Mentha* genus, contain high levels of antioxidants including phenolic compounds, ascorbic acid and carotenoids that can delay or inhibit the oxidation of different molecules [15]; for example, phenolic compounds act as free radical scavengers and inhibit lipid peroxidation [84]. Notably, the oxidation products such as hydroxyl radicals are extremely reactive oxygen species (ROS) that interact with all kinds of biological molecules within their vicinity and cause cellular damages potentially leading to acute or chronic diseases, including cancer [85,86]. Among the several studies carried out on the antioxidant potential of *Mentha* species, Park et al. reported that out of nine *Mentha* species *M. longifolia* was the most effective one, showing a 88.6% antioxidant activity compared to the 93.0% activity of ascorbic acid at a concentration of 100 µL/mL, whereas *M. suaveolens* ‘Variegata’ (pineapple mint) had no antioxidant activity [15]. In contrast, in another study [82] the order of DPPH scavenging activity level was as follows: *M. piperita*, *M. pulegium*, *M. rotundifolia*, *M. spicata* and *M. longifolia*. Numerous factors are implicated in these apparently conflicting results, for example cultivation methods vs wild harvest of plants, type of laboratory investigations or extraction methods, etc. Without a standardized approach it is not possible to define the consistency of comparisons among different works. It was reported that *M. aquatica* has the highest phenolic, flavonoid and tannin contents as well as the highest antioxidant activities [87]. In vitro studies showed that the *n*-butanol soluble fraction derived from a methanolic extract of *M. spicata* at 10 µg/mL exhibited a significant protective activity against DNA damage caused by free ^·^OH radicals [88]. Differently, Bahadori et al. found that *M. longifolia* showed the highest phenolic content and antioxidant activity [55].

Dzamic et al. showed that *Mentha longifolia* essential oil (MLEO) was an effective DPPH free radical scavenger and exhibited scavenging activity in a dose-dependent manner (IC_50_ = 0.66 mL/mL of solution) [89]. Similarly, MLEO reduced DPPH radicals to their neutral DPPH-H form (IC_50_ = 10.5 μg/mL) [90]. Moreover, it has been observed that extract of naturally dried *M. longifolia* had higher content of phenols (113.8 mg GAE/g) and flavonoids (106.7 mg RTE/g) than the laboratory oven-dried samples. Similarly, a higher antioxidant activity, in terms of ferric reducing power and DPPH scavenging, was also reported for the naturally dried extracts (2.76 mmol Fe^2+^/mg and EC_50_ = 0.02 mg/mL) compared with the laboratory oven-dried samples (1.13 ± 0.11 mmol Fe^2+^/mg of dry extract and EC_50_ = 0.03 mg/mL) [91]. This suggests that processing of *Mentha* plants under appropriate conditions is a key step to retain their maximum antioxidant value. In another study, the superoxide radical scavenging activity of different solvent fractions of *M. spicata* were investigated, showing that the ethyl acetate and aqueous fractions of ethanol extract of *M. spicata* had higher superoxide radical scavenging among others [81]. According to Anwar et al., MLEOs of different chemotypes, harvested in different regions of Saudi Arabia, exhibited a reasonably high extent of DPPH free radical scavenging that was mainly correlated to the variable polyphenols and carvone contents of the tested oils [4]. Saba and Anwar evaluated the effect of harvesting regions on physico-chemical and biological attributes of supercritical fluid-extracted spearmint (*Mentha spicata* L.) leaves EO [92]. The researchers noted that the oils tested effectively scavenged DPPH free radicals as well as inhibited linoleic acid peroxidation depending upon variable contents of total phenolics and flavonoids. In another study carried out by Ed-Dra et al., *Mentha suaveolens* EO showed a significant ferric reducing antioxidant potential and DPPH free radical scavenging activity [93]. Dhifi et al., reported that essential oil of *Mentha spicata* showed high activity against *S. epidermidis* and *S. aureus*, as well as Gram-negative cells of *Salmonella* spp. and *E. coli* [94]. Sokovi’c et al. also reported that menthol was more active than other compounds extracted from tested plants: linalyl acetate, limonene, β-pinene, α-pinene, camphor, linalool and 1,8-cineole [95]. Singh et al., reported the antioxidant activity of *M. piperita* measured by evaluating its antioxidant capacity, DPPH free radical scavenging activity and reducing power. A chloroform extract and peppermint oil showed antioxidant potency of about 90% and minimum activity was recorded for the aqueous extract. The IC_50_ (μg/mL) of peppermint oil by using DPPH scavenging method was found to be 15.2 ± 0.9 [96].

Though it is known that the extraction method, the solvent used and the extracted fractions analyzed have direct effect on the composition and ratio of compounds, the results of different studies showed that *Mentha* species, given their significant antioxidant activities, can be confidently used in pharmaceuticals, food and cosmetic productions when antioxidant effects are needed.

### 4.2. Antibacterial Activities

Infectious diseases are considered as one of the growing concerns in medical science worldwide [97] and, in the latter respect, the microorganisms such as pathogenic bacterial and fungal strains are major agents of infectious diseases. Importantly, most such microorganisms have the ability to survive under harsh environmental conditions and can develop multidrug resistance. Regardless of the availability and use of effective antibiotic drugs, a range of multidrug-resistant strains of microorganisms have been posing health threats [98]. Moreover, in the developing and underdeveloped countries, synthetic drugs are not only expensive and available in limited amount to treat infectious diseases, but they are often under the standard requirements and exhibit the lowest and/or side effects. Therefore, there is the need to search for novel and safer natural antimicrobial agents to control and fight against microbial infections [81,99]. Plant-based drugs and phytomedicines not only act as natural remedies to treat different diseases, but also serve as prototype to develop novel, safer, and effective modern medicines. The antimicrobial activities of *Mentha* EOs have mainly been attributed to volatile bioactives such as oxygenated monoterpenoids along with monoterpene hydrocarbons (MHs) and sesquiterne hydrocarbons [81]. *Mentha* EOs are found to exhibit antibacterial activities against pathogenic bacteria including both Gram- negative and Gram-positive, such as *Pseudomonas aureus*, *Bacillus subtilis*, *Escherichia coli*, *Pseudomonas aerogenosa*, *Serratia marcesens*, and *Streptococcus aureus* [81,84]. Saba and collaborators reported the *Mentha piperita* broad spectrum antibacterial activity against bacterial strains, such as *E. coli*, *Salmonella typhius*, *B. subtilius*, *S. aureus*, *P. aeruginosa*, *Staphylococcus epidermititis*, and *Klebsiella pneumonia* [92]. According to another study, MLEO exhibited strong antibacterial effects especially against Gram-negative strains including *P. aerginosa*, *E. coli*, and *S. enteric* [100]. Mahady et al. found that methanol extract from peppermint was weakly active against 15 strains of *Helicobacter pylori* with minimum inhibitory concentration (MIC) in the range of 25–100 μg/mL [101]. However, the reported antibacterial effects of peppermint oil against different bacteria are random type, possibly due to difference of the plant varieties and bacterial strain used and/or testing conditions. In Anwar et al.’s investigation, MLEOs showed a natural antimicrobial effect against different strains of bacteria, but their activity varied with respect to the concentration of volatile chemical constituents [81]. The antibacterial potential of the oils tested was comparable with synthetic drug and its major chemical constituent, carvone. In another report, the EO isolated by supercritical fluid extraction (SCFE) from *Mentha spicata* leaves also revealed good antibacterial potential against selected strains of bacteria such as *E. coli, S. aureus, B. aereus, B. pumilis, B. subtilis, P. aeruginosa*, and *S. poona* [102]. The authors noted that the antibacterial activity of the tested oils varied with respect to the oil composition depending on harvesting regions; however, it was relatively comparable with positive control (synthetic drug). Generally, the oil extracted from drought stressed spearmint populations showed greater antimicrobial activity and those from colder/hilly region exhibited a greater antioxidant activity and total phenolics and flavonoids content [103]. Similarly, Ed-Dra et al. evaluated the antimicrobial effect of *Mentha suaveolens* EO against pathogenic bacteria, showing that the EO of this species has antibacterial effect against Gram-negative and Gram-positive bacteria and hence could be used as a food additive to enhance the shelf- life of food products [93].

Several studies showed that essential oils exerted their antibacterial effects via disrupting the structure of membranes, resulting in loss of integrity and increased cell permeabilization [103,104,105]. The hydroxyl group in phenol compounds was supposed to have a significant role in the antimicrobial activity of essential oils [106,107]; treatment of *E. coli* cells by phenolic compounds caused surface blebbing and inhibition of RNA and protein synthesis [108]. Over the last years, the antimicrobial, antifungal, antiyeast and antiparasitic activities of essential oils and extracts of *Mentha* species have been studied. Stanisavljević et al. prepared the essential oils from *M. longifolia* using three different methods including the natural way, in the laboratory oven (45 °C) and in the absorptional low-temperature condensational drier (35 °C). They showed that the essential oils obtained by the last method had the strongest antimicrobial and antifungal effects, while the essential oils prepared in natural way showed the strongest antioxidant activities [109]. Nikšić et al. reported that essential oils from *M. longifolia* exhibited significant antibacterial effects on gram-negative bacteria including *E. coli*, *P. aeruginosa* and *S. enterica* [90]. In another study, Gulluce et al. showed that the volatile oils of *M. longfolia ssp. longfolia* revealed strong antimicrobial activities against 15 bacteria, 14 fungi and four yeast species [110]. Samber et al. [111] showed that menthol, one of the main components of *Mentha* species essential oils, inhibited the H^+^-ATPase pump and intracellular acidification in *C. albicans* cells. They also reported that menthol inhibited ergosterol biosynthesis pathway and influenced the membrane fluidity and integrity, thus leading to leakage of the intracellular contents [111]. The volatile oil of *M. spicata* showed remarkable antibacterial activity on different *Xanthomonas* strains, and also showed mycelium growth inhibition effect on *A. solani*, *R.solani*, *V. dahlia* and FORL (*F. oxysporum* f. *spradicis-lycopersici*), in a dose-dependent manner [112].

It was reported that ethanol extract of *M. arvensis* induced the generation of ROS in *A. baumannii* cells in a dose-dependent manner, triggering cell membrane damage and protein leakage from the treated cells in a dose-dependent and time-dependent manner [113]. Moreover, structural equation modeling (SEM) visualizations indicated that increasing the extract concentration could provoke considerable cellular damages and morphological changes, consistent with ROS generations and protein leakage [113]. In two different studies, the antibacterial and antiadhesive activity of *M. piperita* ethanol extract on beverage spoilage bacteria was investigated on some acetic acid bacteria of *Asaia* genus, showing that the mint extract caused a reduction in cell adhesion of *As. Bogorensis* and *As. Lannensis* and biofilm formation through its antibacterial activity and its direct effects on extracellular substances [112,113]. Husain et al. reported that treatment of *P. aeruginosa* PAO1 by MPEO resulted in a decrease in the production of: LasB elastase (the major virulence factor); pyocyanin (a toxin produced and secreted by *P. aeruginosa*) up to 85%; exopolymeric substance (EPS); β-galactosidase activity up to 54.5%; acyl homoserine lactone (AHL) levels that regulate virulence factors and biofilm formation in *P. aeruginosa* and *Aeromonas hydrophila* [114].

In another study, *M. piperita* L. leaf extract showed stronger activity against Gram-positive *Staphylococcus aureus*, *Bacillus subtilis* than against Gram-negative *Escherichia coli* [115]. Another study conducted by Laggoune et al. found the *E. coli* and *Proteus mirabilis* strains were sensitive to *Mentha spicata* [116]. Antibacterial effects of peppermint water extract were also observed against *Pseudomonas aeruginosa* and *Serratia marcescens* [117]. Golestan et al. observed that *M. spicata* EO had the highest inhibition activity against *S. aureus* and *Clostridium perfringens* [118]. In another study, Zaidi and Dahiya [119] determined the antimicrobial activity of *Mentha spicata* and *Mentha piperita* EOs against 11 bacterial and four fungal clinical isolates. They reported maximum zone of inhibition of 21±0.09 mm against *S. aureus*, with *Mentha spicata* and 19.2±0.07 mm with *Mentha piperita* [119]. Singh et al. [96] assessed the antibacterial activity of *M. piperita* oil and different extracts (petroleum ether, chloroform, ethyl acetate, ethanol, aqueous) using the agar well diffusion method. Gram positive bacterial species (*S. aureus* and *S. pyogenes*) were tested sensitive to peppermint essential oil with the inhibition zone 17.2 and 13.1 mm, respectively. The inhibition zone for Gram negative bacteria ranges from 5.1 to 12.4 mm [96].

*Mentha* component activity against multiple strains of bacteria mentioned above will have a pronounced impact in the future production of novel plant-derived drugs and in food storage/protection. However, the antimicrobial effect needs to be better elucidated considering that it might be due to the occurrence or cooccurrence of bioactives such as luteolin, rosmarinic acid, caffeic acid, gallocatechin, epigallocatechin gallate and catechins, menthone, isomenthone, and hexadecanoic acid in this species.

### 4.3. Antifungal and Antiyeast Activities

Fungal diseases are a severe health issue especially in subtropical and tropical regions of the world [120]. Due to microbial resistance against common antifungal drugs, there is an urgent need for discovery and development of novel plant-based natural antifungal agents [121]. Besides antibacterial activity, *Mentha* species have also been investigated as a potential source of antifungal agents to control pathogenic molds [92]. Antifungal activity of *M. spicata* was studied by Nosrati et al. and it was found that the EO significantly restricted the mycelia growth of *Fusarium oxysporum* sp. in a dose-dependent manner [122]. The antifungal potential of EO of four *Mentha* species, including *M. arvensis*, *M. piperita*, *M. longifolia*, and *M. spicata*, was evaluated by Hussain et al. [65]. The results of the latter research from the disc diffusion assay followed by MIC revealed that *M. arvensis* exhibited maximum antimicrobial activity with larger IZ (14–33 and 16–30 mm) and smallest MIC values (20.0–330.3 and 56.2–139.0 μg/mL) against selected strains of bacteria and fungi, respectively. *Mentha piperita, M. longifolia,* and *M. spicata* also exhibited a remarkable antimicrobial potential with IZ 15–20, 16–31, and 12–29 mm and 11–32, 19–30, and 16–29 mm against selected strains of bacteria and fungi, respectively. In another report, *M. spicata* EO was shown to be a good natural antifungal agent against pathogenic molds such as *Mucor mucedo*, *Aspergillus niger*, *Fusarium solani*, *Botryodiplodiatheobromae*, and *Rhizopus solani* [123]. Furthermore, recent report on the antifungal activity of chitosan in both its natural and nanoparticle forms revealed that incorporation of mint extract into chitosan nanoparticles resulted in increased antifungal effects against mycelium growth of *A. niger* [124]. The latter finding supports the potential uses of *Mentha* extracts and oils as antifungal agents in nanocapsulation of different food bioactives such as biopeptides. Moreover, the extract from *M. longifolia* (5 μL/mL) showed an effective fungicidal potential against *Aspergillus, Fusarium, Penicillium funiculosum*, and *Trichoderma viride* [89]. The most sensitive strains were *Cladosporium fulvum, Penicillium ochrochloron*, and *Cladosporium cladosporioides* where a concentration of as small as 2.5 μL/mL was found to be lethal [89]. Similarly, Moghtader evaluated the antifungal activity of MPEO against *A. niger* and reported that the oil possessed stronger antifungal activity than standard antibiotic, gentamycin [125]. The antifungal activity of *M. piperita* oil can be mainly ascribed to high content of oxygenated monoterpenoids, such as menthone and menthol. The broad-spectrum antifungal activity of *Mentha* EOs can be linked to the presence of major chemical constituents such as menthone, menthol, piperitenone oxide, and carvone [89].

The essential oil of *M. longifolia* showed antifungal activity against *C. albicans* at a concentration of 7120 mg/mL [126]. *Mentha x piperita* (Mentha of Pancalieri) EO exerted the most remarkable antifungal activity against *Cryptococcus neoformans* and showed non-negligible activity against *Candida krusei* and *C. glabrata* displaying the lowest minimum inhibitory concentration (MIC) and minimum fungicidal concentration (MFC) values ranging from 0.06–0.125%, *v*/*v* [127].

*Saccharomyces cerevisiae* has been frequently used as a model organism to study the anti-yeast activities and investigate the mode of actions of different compounds [128,129]. It was shown that treatment of *S. cerevisiae* cells with MPEO caused the increase of intracellular ROS species, mtDNA damages, mitochondrial fragmentations, formation of petite mutants because of loss of respiratory chain function and chromatin condensation (with no effect on plasma membrane) [130]. In contrast, in another study, plasma membrane rupture, its depolarization and increased permeability was reported [131], thus confirming that different methods can lead to different results.

### 4.4. Antiviral Properties

Viral dependent infectious diseases are a dilemma in medical sciences. Many viruses are resistant to different therapies due to their adaptable lifestyle and, therefore, the development of long-term effective antiviral chemotherapeutic agents is a very challenging task. Natural products have always been deemed the best source for isolating chemically diverse new lead molecules and served as a platform for the future development of potent and safer antiviral agents. Preliminary evidence suggested that the main peppermint oil component, menthol, might have served as natural antiviral agent and protected against *Herpes simplex* [132]. As far as antiviral potential of *Mentha* plants is concerned, it is reported that *Mentha spicata* and *Mentha spicata* essential oil (MPEOs) contain some compounds acting as antiviral agents [133]. Appropriate studies showed that phenolic constituents such as rosmarinic acid, luteolin, and phytol present in the extracts of *Mentha spicata* are effective for their antimicrobial and antiviral actions [134]. Yamasaki et al. reported that water soluble extract (16 μg/mL) of *M. piperita* exhibited strong antihuman immunodeficiency virus-1 (HIV)-1 activity in MT-4 cells assay [135]. Similarly, methanolic and ethyl acetate extract of *M. longlifolia* showed significant inhibitory effects against human HIV-1, and ethyl acetate extract exerted its anti-HIV-1 effects via inhibiting the reverse transcriptase enzyme [136]. Water-soluble (polar substances) extract of *M. piperita* also exhibited inhibitory activity against HIV-reverse transcriptase. MPEO was also reported to have direct virucidal activity against *Herpes simplex* virus type 1 (HSV-1) and reduced plaque formation effectively [137].

### 4.5. Anticancer Activity

Cancer is a multistep disease that is characterized by out-of-control multiplication of cells and currently it is a challenging health problem worldwide [138]. According to the recent report of the World Health Organization (WHO), there are more than 18.1 million new cases and 9.6 million cancer deaths in 2018 [139]. It has been reported that 10–70% cancer mortality may be related to the diet, and about two-third of human cancers would be avoidable by choosing an appropriate lifestyle [140,141]. Cancer has been a continuing health problem in the medical sciences globally, with a lot of developments achieved in the treatments of this chronic disease via use of different preventive and curing therapies. The disease is characterized by the continuous multiplication of the human body cells, with the inability to be controlled or stopped, and the consequent formation of malignant cell tumors with the potential to be metastatic [142]. Current treatments include surgery, radiotherapy, and chemically derived drugs. Treatments such as chemotherapy can put patients under a lot of strain and further damage to their health, and therefore there is a focus on using alternative treatments and therapies against cancer [143]. Herbal medicines have been used for many years in developing countries as the primary source of medical treatment [144,145], and recently research is being directed to investigating the potential uses of terrestrial plant extracts as reducing and capping agents for the preparation of nanomaterial-oriented drugs for cancer control [146]. Many plant species have been screened by the researchers for their anticancer potential, and several of them are also employed in herbal medicine for cancer relief [147]. Plant potential in cancer prevention and treatment has received much attention, due to the presence of phytochemicals [141]. Although some plant-originating anticancer drugs including vinblastine, vincristine, vinorelbine, vindesine, estramustine, taxol and colchicine have been used in clinic [148], the ongoing clinical trials on the application of plant-based nutritional supplements and diets aimed to prevent cancers are still few [149].

The Labiatae family includes some of the most acknowledged and famous medicinal plants, since numerous studies have reported the cytotoxicity effect of *Mentha* species. For example, Rahimifard et al. [150] studied the cytotoxicity effects extracts and essential oils of five *Mentha* species on HeLa (human malignant cervix carcinoma), Hep2 (human laryngeal carcinoma), and Vero (green African monkey kidney) cell lines. They found that EOs showed greater cytotoxicity potential than the extracts and were more toxic in Hela cell line (IC_50_ ≤ 42.3 μg/mL), while the extracts were more toxic against Vero cell line (IC_50_ ≥ 94.3 μg/mL). Hep2 cells showed less sensitivity than the other two cell lines [150]. Differently, aqueous extract of *M. spicata* showed cytotoxicity effect on U937 leukemia cells (IC_50_ = 4.8 mg/mL) [151]. The methanolic extract of *M. longifolia* at a concentration >0.5 µg/µl demonstrated antiproliferative effects in adrenocortical tumor cell lines, with modulating activity on MAPK and PI3k/Akt pathways [152]. Ohara and Matsuhisa screened 120 medicinal plants for antitumor effects against the okadaic aid (OA) tumor inducer and noted that *Mentha* species were among the most effective in antiproliferative activity (86–100%) [153]. According to Hussain and co-workers, hydrodistilled EOs from four commonly cultivated *Mentha* species such as *M. longifolia*, *M. spicata*, *M. arvensis*, and *M. piperita* exhibited good cytotoxicity against the human breast cancer cell line MCF-7 [123]. Another work screened various species of *Mentha* plant extracts for antitumor activities and found that aqueous extract of *M. pulegium* showed the best antitumor activity (94%), whereas *M. longifolia* extracts exhibited reasonable antitumor activity [154]. Similarly, methanolic extract of *M. longifolia* at a dose of 500 μg/mL resulted in 25% cell death and this cytotoxic effect was dose-dependent, that is, further increase of the extract doses up to 1000, 1500, and 2000 μg/mL produced 25, 50, and 62.5% deaths, respectively [155]. In a recent study, Al-Ali et al. reported that water and methanolic extracts of *M. longifolia* had significant antimutagenic and anticancer activities as depicted by brine shrimp bioassay and Ames mutagenicity bioassay [156]. Furthermore, it was showed that the methanolic extract of *M. pulegium* had no cytotoxic effects, but its essential oil proved to be a potent cytotoxic agent on SKOV-3, HeLa and A549 cell lines [157]. Jain et al. showed that chloroform and ethyl acetate extracts of *Mentha* leaves have significant cytotoxicity in time- and dose-dependent manner on HeLa, MCF-7, Jurkat, T24, HT-29, MIAPaCa-2 cell lines; these extracts also induced G1 cell cycle arrest, apoptosis, upregulation of *Bax* and *p53* genes, some cytokines including TNF, IL-6, IFN-γ, IL-8, and induction of senescence phenotype in treated cells [158]. Silver nanoparticles containing the aqueous extract of *M. pulegium* showed significant cytotoxicity on MCF-7 [159] and triggered caspase 9-dependent cell death in MCF-7 and MDA-MB-231 cells [160]. l-Menthol, one of the main components of *Mentha* species, has multiple applications in traditional medicine. Faridi et al. observed that it modulated tubulin polymerization, induced apoptosis and also suppresses the expression of HSP90 [161]. In their proposed model, the latter authors suggested that l-menthol provoked apoptosis directly via caspase 10 activation and also activated caspase 9 which indirectly triggered caspase 3 and mitochondrial cell death [161]. Perillyl alcohol is a monoterpene isolated from lavendin, peppermint, spearmint and some other plants [162], and several studies showed that this compound could inhibit tumor progression. Reddy et al. reported that perillyl alcohol at 1 g/kg level led to apoptosis induction and significantly inhibited the incidence and multiplicity of the adenocarcinoma of the colon in treated rats [163]. Further studies showed that perillyl alcohol induces G0/G1 cell cycle arrest and apoptosis in Bcr/Abl transformed myeloid cells through inhibition of Ras signaling pathway [164,165], or induction of Bak protein in pancreatic ductal adenocarcinoma cells involved in mitochondrial cell death pathway [166]. Perillyl alcohol also delayed and inhibited tumor formation in non-melanoma model of mouse skin carcinogenesis and inhibited UV-B-induced AP-1 transactivation in cultured human keratinocytes and transgenic mice that expressed a luciferase reporter regulated by AP-1 response element [167]. *M. piperita* extract showed a neuro-protective effect in gamma-irradiated mice [168], and the oral administration of its water extract in papilloma tumors mice (initiated by 7,12-dimethyl benz(a)anthracene, DMBA) reduced the incidence of tumors by 64% and increased the latency period of the appearance of papilloma [169]. 4-nitroquinoline-1-oxide (4-NQO) is a quinoline derivative that has tumorigenic potential through induction of DNA lesions and chromosome damages [170]. It was reported that the chloroform, the hexane, and the ethyl acetate fractions of *M. spicata* reduced the frequency of micro-nucleated polychromatic erythrocytes in mouse bone-marrow induced by 4-NQO, and moreover, treatment of mice with these fractions resulted in a significant decrease in apoptotic cells. Among the latter fractions, the ethyl acetate showed the highest effectiveness against 4-NQO [171]. Ifosfamide is a chemotherapy drug and an immunosuppressive agent that is used to treat many different types of cancers including testicular cancer, bladder cancer, small cell lung cancer, cervical cancer, ovarian cancer and osteosarcoma [59,172]. The exact mechanism of ifosfamide action has not been determined yet, but it reportedly binds to the N-7 position of guanine and results in inter- and intra-strand cross-links in the DNA and cell death [173]. *M. spicata* extract at dose 400 mg/kg showed strong anti-mutagenic effect against clastogenic action of ifosfamide in bone marrow cells and sperm abnormalities [174]. Ethanol extract of *M. arvensis* showed the highest anti-angiogenic effects in the chick chorioallantoic membrane (CAM) assay, followed by methanol and ethyl acetate extracts. Treatment of CAMs with 500 μg of the ethanol extract at 96 h resulted in completely suppression of angiogenesis [175,176]. *M. aquatica* essential oil revealed suppressor activity against skin carcinogenesis by suppression of keratin 14 and COX-2 overexpression in 7,12-dimethylbenz[a]anthracene/12-*O*-tetradecanoylphorbol-13-acetate (DMBA/TPA)- induced two-stage carcinogenesis mouse models [177]. Matrix metalloproteinases (MMPs) are a group of Ca^2+^- and Zn^2+^ -dependent endopeptidases that participate in extracellular matrix remodeling and degradation [178]. They have key roles in numerous basic and fundamental physiological processes including tissue remodeling, angiogenesis, wound healing, and migration and also have significant role in several pathological conditions including cancer progression and invasion [179]. *Mentha* species exhibited modulatory effects on the expression or activity of some MMPs, and Liu et al. reported that 10, 30, and 100 mg/kg of spearmint oil significantly decreased the expression of TNF-α, IL-1β, and MMP-9 in rat lung tissues [180]. Moreover, treatment of Wehi-164 fibrosarcoma cells with aqueous extract of spearmint led to significant reduction in MMP-2/MMP-9 activity in a dose-dependent manner, as there was no detectable activity of MMP-2/MMP-9 in Wehi-164 cells treated with 10 mg/mL of the extract after 24, 48, and 72 h [151]. A phytomics analysis (analysis of primary and secondary metabolites of plant extracts) showed that 500 µg/mL of *M. piperita* extract had significant inhibitory effect on the activity of MMP-1 (84.4%), MMP-8 (80.46%), MMP-13 (91.02%) [181]. Wound-closure assay demonstrated that treatment of HT-29 cells with concentration of 500 μg/mL of spearmint phenolic extract produced a blockade of invasion potential of HT-29 cells, though gelatin zymography experiments showed that the activity of MMP-2 and MMP-9 was not significantly inhibited. Also, spearmint extract led to reduced expression of iNOS (inducible nitric oxide synthase) in the 2,4,6-trinitrobenzene sulfonic acid (TNBS)-induced chronic inflammation rat models [182]. Heat-induced expression of MMP-1, MMP-3, MMP-10, MMP-12, and MMP-13 in human dermal fibroblast was significantly inhibited by 100 μg/mL of apple mint leaves extract (ALE). Also, the activity of p-ERK, p-JNK, p-p38, and the expression of IL-8 were inhibited in a dose-dependent manner in HDF cells treated with ALE [183].

Epithelial-mesenchymal transition (EMT) is a feature of advanced carcinomas, essential for many developmental processes, wound healing, organ fibrosis and also is one of the causes of cancer cell invasion and metastasis [184]. EMT involves loss of epithelial markers including E-cadherin, weakening of cell-cell adhesion, and the expression of some fibroblast markers including vimentin, α-smooth muscle actin (α-SMA), and desmin [185,186]. Moreover, it is well known that adding TGF-β to epithelial cells in culture results in EMT induction in different epithelial cells [187]. Treatment of CCl_4_-induced liver fibrosis rats with MPEO produced a significant reduction in ALT, AST, MDA and NO (nitric oxide) that are triggered by CCl_4_ [188]. In addition, MPEO administration resulted in inhibition of the TGF-β1/SMAD signaling pathway, downregulation of desmin and α-SMA, which are mesenchymal markers, and p53 and upregulation of *CYP2E1* in rats treated with CCl_4_. Nakamura et al. reported that piperitenone oxide, a component of the *n*-hexane fraction of the spearmint extract, induced duct formation in RCM-1 human colon cancer cells, a process that is a considered a differentiation marker, i.e., with potential anticarcinogenic activity [189]. In vitro anticancer potential of methanolic and aqueous extracts of *Mentha arvensis*, *M. longifolia*, *M. spicata* and *M. viridis* at concentration of 100 µg/mL was evaluated against eight human cancer cell lines—A-549, COLO-205, HCT-116, MCF-7, NCI-H322, PC-3, THP-1 and U-87MG from six different origins (breast, colon, glioblastoma, lung, leukemia and prostate) using sulphorhodamine blue (SRB) assay. Methanolic extracts of *Mentha* spp. displayed anti-proliferative effect in the range of 70–97% against four human cancer cell lines, namely COLO-205, MCF-7, NCI-H322 and THP-1; however, aqueous extracts were found to be active against HCT-116 and PC-3. However, essential oil from *M. pulegium* was found to be a cytotoxic agent against human ovary adenocarcinoma SK-OV-3, human malignant cervical adenocarcinoma HeLa and human lung carcinoma A-549 cell lines [157].

*Mentha* species contain numerous bioactive constituents, which have been shown to possess anticancer activity that in turn can act as lead molecule for discovery of new anticancer drugs with additional protective role against different pathogens, as underlined by their antimicrobial properties.

## 5. Clinical Trials

Limited data are available on clinical trials using *Mentha* species in humans [190], and indeed only two works reported the use of *Mentha* species in humans related to cancer. First a randomized, double-blind clinical trial was conducted in 200 patients to determine the efficacy of volatile oils of *Mentha piperita* or *Mentha spicata* in preventing chemotherapy-induced nausea and vomiting (CINV) in four groups, control, placebo, *M. piperita, M. spicata*. The results showed a significant reduction in the intensity and number of emetic events in the first 24 h with *M. spicata* and *M. piperita* in both treatment groups (*p*< 0.05) when compared with the control and no adverse effects were reported. The cost of treatment was also reduced when essential oils were used [191]. The second work is another randomized, double blind placebo clinical trial conducted in 60 patients to evaluate the effects of *Mentha piperita* (and *Matricaria recutita*) on oral mucositis (OM) in patients undergoing hematopoietic stem cell transplantation (HSCT) [192]. OM is one of the most common side effects of intensive chemotherapy in patients undergoing HSCT. Patients who received herbal mouthwash three times daily for 1 week before HSCT showed significant improvements in pain intensity (*p* = 0.009), dryness (*p* = 0.04) and dysphagia (*p* = 0.009), suggesting a therapeutic role for *M. piperita* in OM.

## 6. Adverse Effects of *Mentha* Species

Although medicinal plants such as *Mentha* species are commonly believed to be safe, they are not devoid of side effects that can be severe in some cases. Furthermore, allergic reactions can occur with any natural or synthetic compound in sensitive persons. No chronic toxicity studies in humans are available, therefore toxicity of *Mentha* species are scarcely reported. However, it seems that no adverse effects have been reported after consumption 0.24 mL of pure *M. spicata* essential oil daily for three continuous weeks in two different clinical studies [193,194]. Leaves of *Mentha spicata* are known for its contact allergy such as contact cheilitis caused by its essential oil use as toothpaste flavoring [195]. In addition, MPEO is also associated with adverse effects like vomiting, headaches, flushing, heartburn and nausea [196]. *Mentha piperita* and spearmint tea can deprive the human body of iron and cause anemia if consumed excessively, and carvone and limonene showed to be major allergens [195]. Gürbüz found that pulegone, contained in low concentrations in *Mentha piperita* oil extracts, is hepatotoxic, and Douros et al. also reported the likely liver injury caused by *M. piperita* [197,198]. Other research showed that menthol and pulegone could be toxic compounds; in particular, pulegone and its metabolite menthofuran have been suggested as the hepatotoxic compounds in *Mentha pulegium* and have been also found in smaller quantity in *Mentha piperita* [199]. Notably, inhalation of menthol can cause apnea and larygospasm in sensitive patients and indeed has been reported that mentholated preparation can be involved in nausea, anorexia, cardiac problems, ataxia and other CNS symptoms [200]. *Mentha spicata* extracts displayed toxicity to neuronal cells when applied at concentrations which are one order of magnitude higher than those effective for radical scavenging [201]. The positive correlation between the two aforementioned effects suggests that a higher desirable radical scavenging is associated with a higher undesirable toxicity.

Peppermint oil is contraindicated in obstruction of the bile ducts, gallbladder inflammation, and severe liver failure [199]. The American College of Gastroenterology has recommended reducing the peppermint intake as it is a risk factor for gastroesophageal reflux disease (GERD) and lifestyle changes [202,203]. Further, Zong et al. [204] reported that peppermint essential oil, not only stimulated bile fluid secretion, but might be involved in upregulating the bile acid synthesis-related gene, cholesterol 7α-hydroxylase (CYP7A1), and the nuclear bile acid receptor FXR (farnesoid X receptor) mRNA. In addition, peppermint oil could cause heartburn or perianal irritation, bradycardia and muscle tremor, a hypersensitivity reaction, contact dermatitis, abdominal pain and jaundice in newborn babies [205]. A study on a 58-year-old woman who smoked menthol cigarettes also established that she suffered from gastrointestinal upsets with occasional vomiting, hand tremor, mental confusion and depression which were all ascribed to menthol [206]. Similarly, in another case report, a 40-year-old woman with no history of asthma or any other forms of allergy has shown the symptoms of dyspnea, wheezing and nasal after using menthol containing candies and toothpaste, suggesting the development of classical symptoms of asthma [207]. Menthol administered for 28 days at a dose level (≤ 800 mg/kg) in rats caused hepatocellular changes and pulegone (≤ 160 mg/kg) has been reported as hepatotoxic and neurotoxic. Consequently, pulegone caused weight loss, atonia, decreased blood creatinine, histopathological changes in the liver and also in the white matter of the cerebellum [208]. Menthone (≤ 800 mg/kg orally) on the other hand, dose dependently decreased plasma creatinine, but increased alkaline phosphatase and bilirubin along with liver and spleen weight [209]. Menthone administered to rats at a high dose over 28 days did display some signs of hepatotoxicity and cerebellar histopathology [209]. In a later study examining peppermint constituents for their possible induction of encephalopathy, one-month treatment with limonene (≤ 1600 mg/kg) or 1,8-cineole (1000 mg/kg) produced an accumulation of protein droplets containing α-2µ-globulin in proximal tubular epithelial cells, but no encephalopathy in rats [210]. Peppermint and menthol have both been shown to possess Ca^2+^ channel blocking properties, which might underlie their mechanism of efficacy against irritable bowel syndrome in the clinic [200]. However, in some patients, the use of peppermint is accompanied by oral symptoms like burning mouth syndrome and oral ulceration [203]. Also in this context, direct application of peppermint oil to the chest or nasal area of infants is not recommended due to the risk of apnea, bronchial and/or laryngeal spasms [211].

## 7. Conclusions and Future Perspectives

*Mentha* species have been used in indigenous medicine for many centuries and this review attempts to provide an overview on *Mentha* species’ preventive and curative effects. The essential oils derived from *Mentha* species acts as a good expectorant and further have been used as a folk remedy for respiratory diseases such as bronchitis, sinusitis, tuberculosis and the common cold. *Mentha* species’ exploitation in pharmaceuticals formulations requires further research. Likewise, clinical trials are scarce and intense efforts should be made to confirm the claims of efficacy in humans. However, numerous preclinical works have been performed, underlining the antioxidant, antibacterial, antifungal, anti-yeast, antiviral, and anticancer activity. Indeed, *Mentha* species, and especially essential oils, are used to reduce microbial load, suggesting a strong bactericidal, virucidal, and fungicidal activity. Nevertheless, some adverse effects, such as allergic reactions, vomiting, headache, flushing, heartburn and nausea hepatotoxicity, apnea and larygospasm, neuronal cell damage, may arise due to the presence of some compounds (carvone, limonene, menthol, pulegone) also depending on the *Mentha* extract concentration applied. Moreover, the presence of harmful compounds in plant such as pulegone and menthone can be reduced by oven-drying or cooked before consumption in order to make it safer. In addition, care should be taken when this plant is consumed along with drugs which induce P450 enzymes.

The positive results described in the present review are a key-point of efficacy of *Mentha* species, to such an extent that they can be considered promising natural extracts with a clear application as preservatives, supplements, and antioxidants. This intended role of mint cannot be restricted to food preparation, elaboration, or storage, but should be also figured out as a new tool for pharmaceutical industries.

## Figures and Tables

**Table 1:** Scientific names and common names of *Mentha* species.

Scientific Name	Common Name
*M. aquatica* L.	Water mint
*M. piperita* ‘Lavendula’	Lavender mint
*M. arvensis* L.	Corn mint, field mint, ginger mint, wild mint
*M. canadensis* L.	American wild mint, Canada mint, Chinese mint, East Asian wild mint, Japanese mint, Sakhalin mint
*M. longifolia* L.	Himalayan silver mint, horsemint
*M. piperita* L.	Peppermint
*M. piperita f.* citrate	Bergamotmint, eau de cologne mint, orange mint
*M. pulegium*	Mosquito plant, pennyroyal mint, pennyrile, pudding grass, squaw mint
*M. spicata* L.	Ciudad del Este mint, common mint, garden mint, homegrown mint, lamb mint, mackerel mint, spearmint
*M. suaveolens*	Apple mint, pineapple mint, round-leafed mint, woolly mint
*M. suaveolens ‘*Variegata*’*	Pineapple mint
*M. x piperita*f. *citrate* ‘Chocolate’	Chocolate mint
M. *suaveolens× piperita*	Grapefruit mint

**Table 2:** Main chemical compounds isolated from different *Mentha* species.

Species Name	Essential Oil Components	Other Polyphenol Compounds	References
*M. aquatica* L.	epi-bicyclosesquiphellandrene, 1,8-cineole, menthofuran, β-caryophyllene, limonene, *p*-menthone, β-pinene, germacrene D, α-pinene, α-humulene, δ-cadinene, caryophyllene oxide, viridiflorol, viridiflorol epoxide II, α-cadinol, β-bisabolenol, α-trans-bergamotene, *p*-cymene, borneol, sabinene, β-myrcene, terpinyl acetate, eucalyptol	Rosmarinic acid, lavandulifolioside, rutin-*O*-glc, eriodictyol-*O*-rut, quercetin-3-*O*-soph, verbascoside, caffeic acid	[31,32,33,34,35,36]
*M. arvensis* L.	3-Octanol, fenchone, endo-fenchol, *p*-menthone, iso-menthone, neo-menthol, menthol, epi-bicyclosesquiphellandrene, isopulegone, 1-α-terpineol, pulegone, eugenol, *cis*-jasmone, β-bisabolene, *cis*-3-hexenyl phenyl acetate, β-eudesmol, oxygenated monoterpenes, 1,8-cineole, β-caryophyllene oxide, linalyl acetate, α-phellandrene, terpinolene, limonene, pulegone	Monogalactosyl diglycerides, digalactosyldiglycerides, decyl anhydride, 1-decanol	[17,35,36]
*M. canadensis* L.	Oxygenated monoterpenes, 1-menthol, isomenthone, 1-limonene, menthone, neomenthol, isopulegone, pulegone, linalyl acetate, piperitone	3,4-Dihydro-3,6,7-trihydroxy-2(1*H*)-quinolinone, (*E*)-2-methoxy-2- oxethyl-3-(4-hydroxyphenyl) acrylate, syringic acid, *p*-coumaric acid, esculetin, methyl rosmarinate, nepetoidin B, syringaresinol, methyl ester of caffeoyl glycollic acid, 2″,3″-diacetyl- martynoside and bracteanolide A, *cis*-3-[2-[1-(3,4-dihydroxyphenyl)-1-hydroxymethyl]-1,3-benzodioxol-5-yl]-(*E*)-2-propenoic acid	[17,35,37]
*M. longifolia* L.	τ-Cadinol, γ-cadinene, γ-gurjunene, 1-limonene, piperitone oxide, piperitenone oxide, piperitenone, menthone, borneol, pulegone, verbenone, β-caryophyllene, linalool, 3-tripinolenone, dihydrocarvon, 1,8-cineol, germacrene D, citronellal	Prasterone acetate, sclareol	[38,39]
*M. mozaffarian* L.	Piperitone, 1,8-cineol, linalool, α-terpineol	Piperitenone, pulegone, piperitenone oxide, menthone, *cis*-piperitone epoxide	[38,39,40]
*M. piperita* L.	Oxygenated monoterpenes, menthol, methyl petroselinate, menthyl acetate, isopulegol, pulegone, carvone, menthone, cineole, menthofuran, isomenthone, limonene, β-pinene, β-myrcene, α-pinene, α-thujene, linalool	Riboflavin, *cis*-carvone oxide, caffeic acid, *p*-cumaric acid, ferulic acid, rosmarinic acid, caftaric acid, chlorogenic acid, *m*-coumaric acid, *o*-coumaric acid,	[35,41,42,43,44,45]
*M. pulegium* L.	Piperitone, piperitenone, 4-terpineol, menthone, limonene, naringenin, pulegone, iso-methone	Rosmarinic acid, ellagic acid, caffeic acid, caftaric acid, chlorogenic acid, *m*-coumaric acid, *o*-coumaric acid, *p*-coumaric acid, cryptochlorogenic acid, isochlorogenic acid, neochlorogenic acid, protocatechuic acid	[35,46,47]
*M. rotundifolia* L.	Menthol, menthone, menthyl acetate, menthofuran, piperitone oxide, linalyl acetate, neomenthol, piperitone, isomenthone, 1,8-cineole, linalool, geraniol, myrcene, geranyl acetate, germacrene D, carveol, limonene, rotundifolone, *p*-menthane-1,2,3-triol, D-limonene, piperitol, diosphenol, β-caryophyllene,, germacrene D, calamenene, *trans*-piperitone epoxide, piperitenone oxide, *cis-*piperitone oxide, cyclohexanol, *trans*-sabinene hydrate	Hypericin, apigenin, quercetin, *trans*-cinamaldehyde acid, rosmarinic acid, quercetin3-*O*-galactoside, hydroxybenzoic acid, procyanidin B2	[48,49,50,51,52]
*M. spicata* L.	Carvone, piperitenone oxide, pulegone, 1,8-cineole, limonene, *cis*-piperitone oxide, piperitone, piperitenone, menthofuran, caryophyllene	Rosmarinic acid, salvianolic acids, hydroxybenzoic acids, caffeoylquinic acids, hydroxycinnamic acids, flavanones, and flavones	[53,54,55]
*M. suaveolens Ehrh* L.	Piperitenone oxide, pulegone, trans-caryophyllene, germacrene D, nepetalactone, piperitenone, *cis*-piperitone, limonene, menthone, terpinen-4-ol, *p*-cymen-8-ol, E-hydrate sabinene	4-Hydroxybenzoic acid, vanillic acid, chlorogenic acid, syringic acid, *o*-coumaric acid, *p*-coumaric acid	[56,57,58]
*M. viridis* L.	Carvone, 1,8-cineole, 2-methyl- 5-(1-methylethenyl) limonene	Rosmarinic acid, caffeic acid, luteolin-7-*O*-rutinoside, rosmarinic acid and luteolin-7-*O*-glucoside, 3-*O*-caffeoylquinic acid, 3-acylchlorogenic acids	[59,60,61,62,63,64]

**Table 3:** Chemical compounds in *Mentha* genus and their pharmacological properties.

Pharmacological Properties	Chemical Compounds Responsible for Pharmacological Properties	References
Antioxidant	Ascorbic acid, rosmarinic acid, δ-terpinene, α-terpinene, *p*-cymene, 1,8-cineole, *cis*-carveol, carvone, rosmarinic acid, cynaroside, cryptochlorogenic acid, naringin	[59,65,66]
Antibacterial	Luteolin, rosmarinic acid, caffeic acid, gallocatechin, epigallocatechin gallate, catechins, menthone, isomenthone, hexadecanoic acid, *cis*-carveol, carvone, limonene	[4,65,66]
Antifungal and Antiyeast	Limonene, piperitenone oxide, menthol, menthone, carvone, *cis*-carveol and carvone, piperitone, citronellal, caffeic acid, naringin, cryptochlorogenic acid, rosmarinic acid	[4,65,67]
Antiviral	Menthol, eriocitrin, rosmarinic acid, luteolin 7-*O*-rutinoside, hesperidin, phytol	[4,68]
Anticancer	Eugenol, caryophyllene, t-cadinol, menthone, menthol crotonate, naringin, cryptochlorogenic acid, rosmarinic acid	[69,70,71]

## Data Availability

Not applicable.
